# Caller ID for Risso’s and Pacific White-sided dolphins

**DOI:** 10.1038/s41598-022-08184-2

**Published:** 2022-03-16

**Authors:** Mahdi H. Al-Badrawi, Yue Liang, Kerri D. Seger, Christopher M. Foster, Nicholas J. Kirsch

**Affiliations:** 1 Center for Acoustics Research and Education, University of New Hampshire, Durham, NH 03824 USA; 2 Electrical and Computer Engineering, University of New Hampshire, Durham, NH 03824 USA; 3 Applied Ocean Sciences, Fairfax Station, VA 22039 USA

**Keywords:** Animal migration, Classification and taxonomy

## Abstract

Tracking species with expanding ranges is crucial to conservation efforts and some typically temperate marine species are spreading northward into the Arctic Ocean. Risso’s (Gg) and Pacific white-sided (Lo) dolphins have been documented spreading poleward. Further, they make very similar sounds, so it is difficult for both human analysts and classification algorithms to tell them apart. Using automatic detectors and classifiers on large acoustic datasets would improve the efficiency of monitoring these species. variational mode decomposition (VMD) provides both an easier visualization tool for human analysts and exhibited robustness to background noise while extracting features in pulsed signals with very similar spectral properties. The goal of this work was to develop a new visualization tool using VMD and a statistics-based classification algorithm to differentiate similar pulsed signals. The proposed VMD method achieved 81% accuracy, even when using audio files with low SNR that did not have concurrent visual survey data. While many dolphins whistle, pulsed signals are one of the more useful vocalizations to use in detection and classification because of their species-specific acoustic features. Automating the VMD method and expanding it to other dolphin species that have very similar pulsed signals would complement current detection and classification methods and lead to a more complete understanding of ecosystem dynamics under a changing climate.

Marine mammals shifting their habitat ranges may be because of climate change and these shifts can be monitored acoustically by placing underwater recorders throughout the ocean to listen for and track their vocalizations^1–3^. Such acoustical surveys are a complementary monitoring tool to visual surveys because, unlike visual methods, they can collect data overnight and year-round, even in harsh conditions and remote locations^4^. Downsides to acoustical surveys include a lack of concurrent visual observations of animals and the need for many hours of manual analysis—the latter of which this new detector/classifier method aims to expedite. Because of spectral similarity, analyzing dolphins’ clicks, buzzes, burst pulses, and pulsed calls (henceforth called “pulsed signals”) from these acoustical surveys is a challenging task. But being able to use pulsed signals to differentiate between species is an important analysis capability for monitoring species as their populations rebound or decline, or as they adjust to climate change or respond to anthropogenic noise.

The Risso’s dolphin (Grampus griseus, or “Gg”) and Pacific white-sided dolphin (Lagenorhynchus obliquidens, or “Lo”) pulsed signals are a good case study for tracking effects of climate change because they have recently been documented expanding northward into the Bering and Chukchi Seas^2^. Their pulsed signals were recorded in the Arctic Ocean starting in 2009 in areas where they were previously presumed to be extralimital when visually spotted^5^. Between 2009 and 2016, the presence of their pulsed signals coincided with warmer surface temperatures in the Gulf of Alaska (during years with a negative Pacific Decadal Oscillation (PDO)) and a Bering Sea Cold Regime^2^. If Gg and Lo are following prey northward, this combination of the PDO and Cold Regime can foster greater food supply in the pelagic zone^2^ where they both hunt. With climate change, the prevalence of the Bering Sea Cold Regime decreases^6^, the PDO cycle weakens and shortens^7^, and the Arctic Ocean contains less ice^8^, so it will be important to monitor whether these species become more present in the Arctic Ocean in the future. Because Lo and Gg do not have the same diet^9–12^, differentiating between their pulsed signals to track their presence in the Arctic Ocean would help us better understand problems like the effects of climate change on marine mammals, the effect of new players in the Arctic food web, and properly calculating maximum sustainable yield for fisheries management.

Gg and Lo pulsed signals are also a good case study for creating classification algorithms because they have similar spectral features, are used more often than whistles^13^, and are largely above the human hearing range so aural analysis is not possible. While there are other delphinid species that produce pulsed signals in similar bandwidths to Gg and Lo, like killer whales and long-finned pilot whales^14^, these clicks are usually defined by peak and centroid frequencies instead of by peak and notch patterns. The first study to define different peak and notch patterns in the pulsed signals of Lo and Gg was done by Soldevilla et al.^15^. They also measured the statistical differences between Gg and Lo peaks and notches using Gaussian mixture models. Their work was successfully used to associate click types with behaviors in a subsequent study^16^. This instilled confidence that the distinctive peak and notch patterns of the two species are sufficiently stereotyped over time for use in the new method presented here. Since only the overall peak frequency of pulsed signals is normally reported in acoustic feature sets for classification use, any new algorithm used for Gg and Lo would need to handle multiple peak frequencies.

Previous classification work on pulsed signals includes a range of approaches from classical signal processing to machine learning techniques. FFT-based classifiers, such as short-time Fourier transforms or Wigner–Ville transforms^17^, have deteriorating performance with poor-quality (low SNR) files and are computationally expensive^18^. A more efficient FFT method using a two-stage classifier with the cepstrum has classified small dolphins, killer whales, pilot whales, sperm whales, and three species of beaked whales^19^. However, the bandwidths and frequency patterns of these species’ clicks and tones are well-documented in the literature. They are also more distinctive from each other than the pulsed signals are between Gg and Lo.

Gaussian mixture models (GMMs) have some success distinguishing between Lo clicks and burst pulses, common dolphin whistles, and bottlenose dolphin whistles^20^. The Lo pulsed signals were likely differentiated well because they were the most different from the other species in the dataset^20^ and were not tested against Gg pulsed signals. Gg pulsed signals were included, though, in a deep network study recently^21^. Other strategies like combining wavelets and neural networks have differentiated between sperm whale and long-finned pilot whale clicks^22^. A viable alternative to the wavelet transform for odontocete clicks is the Hilbert–Huang transform (HHT)^23^. Empirical mode decomposition (EMD) is the key part of the HHT and it performed well in differentiating between many tonal baleen whale signals^24^. EMD performed less well when applied to pulsed signals, but its advanced version—variational mode decomposition (VMD)—performs much better for feature extraction in pulsed signals. These mode decomposition techniques act like filter banks that break down signals into finite sets of components called intrinsic mode functions (IMFs), effectively sorting a waveform into its most to least powerful bandwidths.

Our proposed *Bayesian VMD Method* includes (1) a detector using proven FFT techniques to find signals in an audio file, (2) a VMD algorithm to extract acoustic features from the detected snippet of sound, and (3) a Bayesian classification weighting system to determine whether Lo or Gg most likely produced the detected sound. The *Bayesian VMD Method* capitalizes on the *VMD-gram*, which is a visualization of VMD IMFs in the time-frequency plane via the Hilbert spectrum. Like the Wigner plot^25,26^ in PAMGuard that makes it easier for a manual analyst to recognize beaked whale upsweeps, the *VMD-gram* can be used in addition to or in place of the spectrogram for manual analysis of pulsed signals with similar peak and notch patterns.

The *Bayesian VMD Method* has two advantages. First, it inherently denoises the data while decomposing it. Software like PAMGuard uses a multiple-phase process to denoise data with its whistle and moan detector^27^. But, any of the denoising steps that aim to remove mechanical pulses may also remove biological pulsed signals. Therefore, the overall framework that the *Bayesian VMD method* came from was designed to detect instead of discard broadband pulsive noises in the detection phase, then parse them into a separate category in the classification phase^24^. With this denoising, the peak and notch patterns are more noticeable to the human eye in the *VMD-gram*^28^ than if they were displayed in a standard FFT spectrogram. This means the *VMD-gram* can have cleaner acoustic features extracted from it, providing cleaner input to a classification algorithm. Second, the *Bayesian VMD Method* uses a probability summation to calculate whether the pulsed signals are more likely Lo or Gg based on parameters set by Soldevilla *et al*.^15^. The probability summation quantifies the relative strength of each classification like in the BANTER software^13^. The likelihood comparison makes it possible to decide the category of the pulsed signal even if some peaks or notches are absent.

The goals when creating the *Bayesian VMD Method* for pulsed signals included:

- using only pulsed signals to distinguish two species in a passive acoustic dataset since not all delphinid species whistle, but they do all click;
- de-noising the data inherently and adaptively to avoid separate denoising steps and to adjust to fluctuating background noise conditions in a file or a dataset;
- testing the Method on poor-quality recordings instead of discarding them, because sparse datasets need to involve as many data points as possible; and
- achieving accuracy levels like previous studies to build a trustworthy product for researchers by demonstrating successful classification with the worst-case scenario.

## Results

### Proposed detector and classifier accuracy

Beginning with ninety audio files that were 4.5 s long, automated detection and manual analysis by an expert identified 174 distinct signals that were labeled as either Lo or Gg. These 174 signals plus other pulses from noises like a mooring chain, a pinger, and unknowns contained 1730 groups of energy peaks made up of 4815 individual energy peaks. Our FFT detector began to group energy peaks with an inter-pulse interval threshold of 10 ms for all files, but 100 ms threshold performed better when click trains were in the file. PAMGuard’s click detector does not do a similar grouping, so the manual analysis had to include a count of individual energy peaks for us to best compare it to our FFT detector. The settings in PAMGuard’s click detector were explored to generate the best detection rate. The best settings were the Ishmael Energy Sum with 20–50 kHz bounds and 10 dB peak detection thresholdwith a 20 kHz high pass filter. The EMD detector from previous work 38 detected 977 of the 1730 energy peak groups, achieving an accuracy of 56.47%. To improve upon this result, we switched to an FFT detector based on PAMGuard’s method that detected 1542 of the 1730 energy peak groups, scoring an accuracy of 89.13%, while PAMGuard’s click detector detected 3876 of the 4815 energy peaks, scoring an accuracy of 80.5%. Scoring an accuracy at least as good as PAMGuard while using inspiration from the authors’ previously published methods made us confident in continuing to the next step: classifying the detected signals.

The basis of using VMD in a classifier was due to the fact that previous work^24^ that used EMD failed to separate Gg from Lo pulsed signals. Both species’ pulsed signals resulted in “EMD identities” of the label “[1, 2]”. For the sake of comparison, though, precision, recall, and accuracy for how the old EMD classifier performed are provided in Table 1. Specifically, 60 of the 84 (71.43%) Gg pulsed signals were correctly classified as the EMD identity [1, 2] and 60 of the 90 (66.67%) Lo pulsed signals were also correctly classified as the EMD identity [1, 2]. Pulsed signals that were classified as EMD identities other than [1,2] came from low SNR files or were missing key frequency content. Therefore, with 60 correct [1, 2] classifications from known Lo signals and 60 correct [1, 2] classifications from known Gg signals, the chance of guessing which species the signals were actually from was a coin toss: 50%. Since some signals were false negatives, accuracy would be worse than a coin toss at 34.48% for each species.

The new classifier using a VMD feature extractor was able to separate Gg from Lo pulsed signals and the results achieved 81.03% overall accuracy (for both Lo and Gg) in contrast to 72.99% overall accuracy for the classifier when using FFT feature extractor results (Table 1). As for the performance of the *Bayesian VMD Method* on each dolphin species, it achieved 88.89% recall on Lo and 72.62% recall on Gg as compared to the FFT feature extractor achieving 72.22% and 73.81%, respectively. The *Bayesian VMD Method* achieved 77.6% precision on Lo and 85.91% precision on Gg, as compared to the FFT feature extractor achieving 74.71% and 71.26%, respectively (Table 1). The FFT feature extractor method slightly outperformed the *Bayesian VMD Method* only for recall with Gg, but otherwise the *Bayesian VMD Method* outperformed the FFT feature extractor method (Table 1). The *Bayesian VMD Method* was better able to identify Lo signals and its accuracy to identify Gg signals is also better.

**Table 1:** Precision (**P**), recall (**R**), and total accuracy (**A**) of three classifiers for Gg and Lo: the EMD classifier^24^, the FFT-based features extractor classifier, and the VMD-based features extracted classifier.

Class	EMD			FFT-based			VMD-based		
	P %	R %	A %	P %	R %	A %	P %	R %	A %
Gg	50	71.43	34.48*	71.26	**73.81**	72.99	**85.9**	72.62	**81.03**
Lo	50	66.67		74.71	72.22		**77.6**	**88.89**	

*Denotes accuracy for either Lo or Gg classifications separately.

### The *VMD-gram* visualization tool

**Figure 1:**
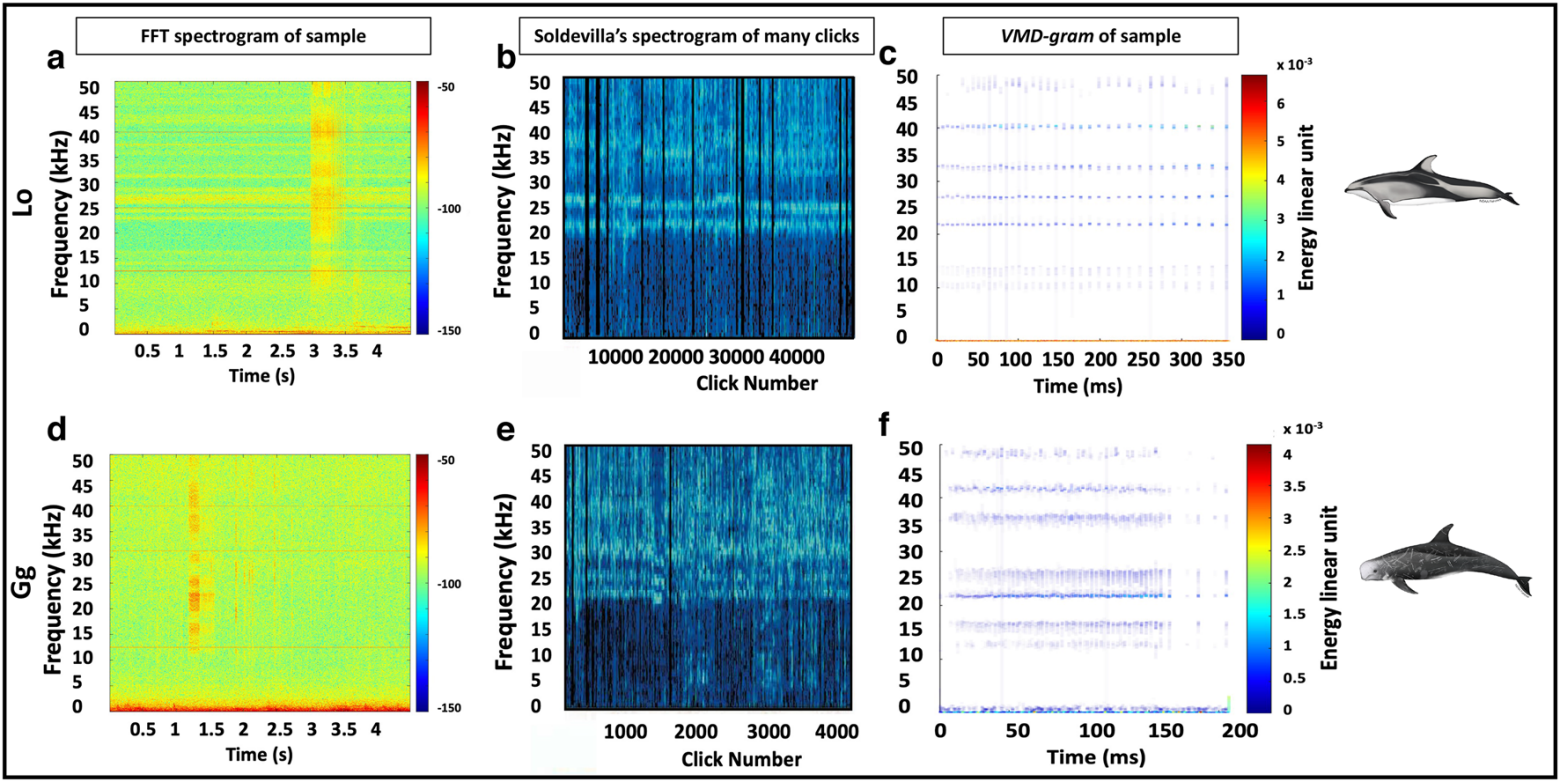
Three visualization tools for delphinid clicks. FFT spectrograms of one sample of a Pacific-white sided dolphin pulsed signal (**a**) and Risso’s dolphin pulsed signal (**d**) compared to the FFT spectrograms of thousands of clicks from each species by Soldevilla et al. (2008) (**b, e**) compared to the *VMD-grams* of the segments with pulsed signals (**c, f**). Ease of visualizing the peak and notch patterns for manual analysis is more evident in the VMD-gram than in the FFT spectrogram.

The *VMD-gram* is the visualization tool that made manual analysis of peak and notch patterns easier to compare to those published in Soldevilla *et al*. (2008). The noise reduction from the FFT spectrogram to the *VMD-gram* can be seen in Fig. 1. Compared to an FFT-gram (a.k.a. a traditional spectrogram), electrical noise bands have largely been eliminated and the energies that are non-stationary (like those produced by animals) are emphasized. The VMD-gram is obtained by applying the Hilbert transform to the VMD output components. The resulting instantaneous energies and frequencies are transformed into a sparse time-frequency matrix^29^. The sparse matrix representation of the *VMD-gram* makes it easier to visualize.

Processing a set of *VMD-grams* is much quicker, by hand, than by squinting at or zooming into spectrograms. For any species identification that relies on frequency banding patterns, the *VMD-gram* accentuates those compared to a spectrogram. The experience of manually analyzing these *VMD-grams* is similar to the Wigner Plot^25^ now used in PAMGuard to quickly identify beaked whale upsweep pulses.

Once the *VMD-gram* is made, the acoustic features that can be extracted from it are also largely void of background noise and peaks and notches become more apparent, making features to feed into classification algorithms more apparent. As an image file, it is also applicable as input to machine learning algorithms such as neural networks.

### The good, the bad, and the ugly

**Figure 2:**
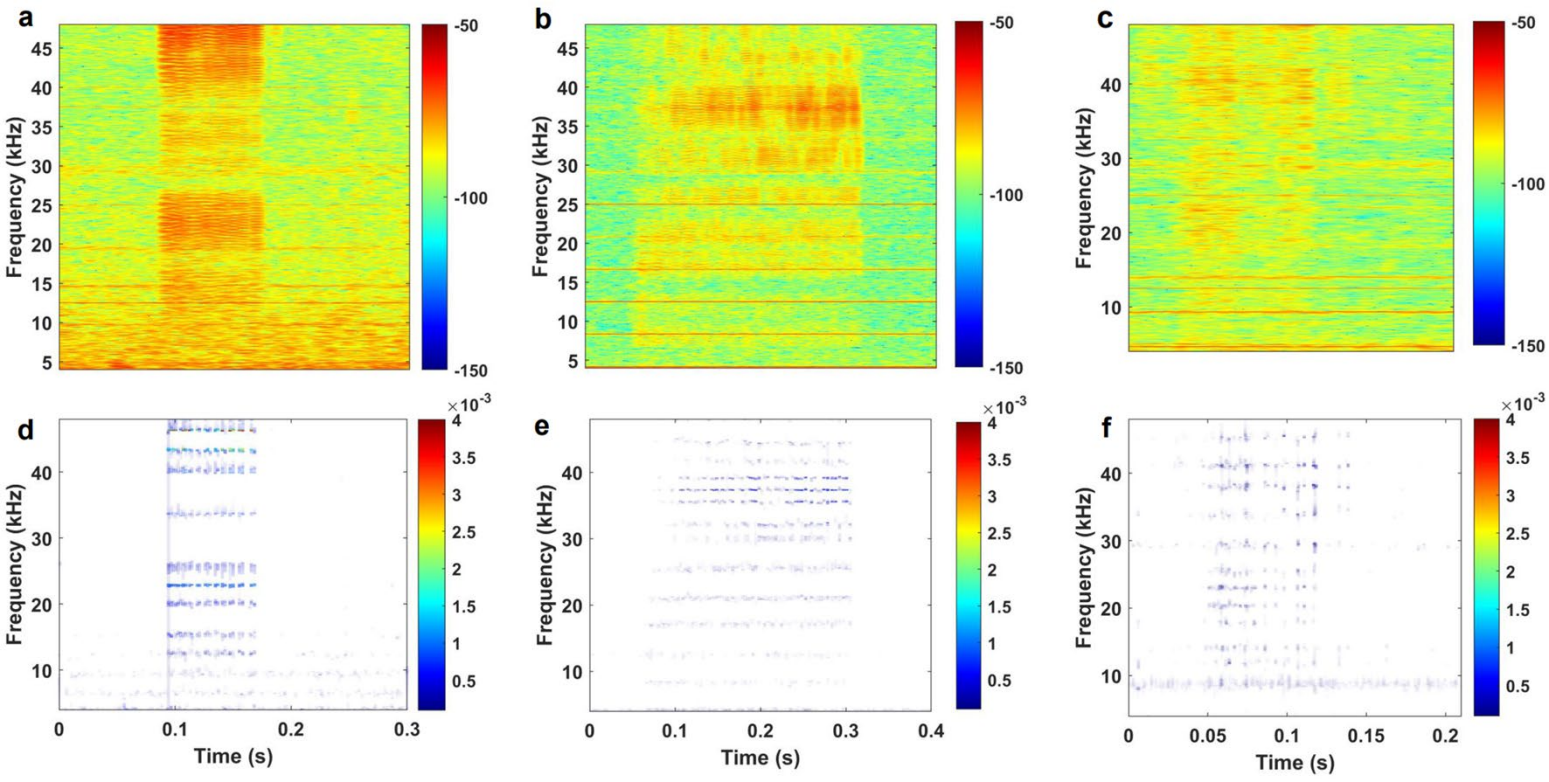
Spectrograms and *VMD-grams* of various data qualities. “Good” (a, d), “bad” (b, e), and “ugly” (c, f) data sample spectrograms (**a**–**c**) compared to their *VMD-grams* (**d**–**f**). “Good” means SNR $>6$ dB and few electrical noise bands; “Bad” means SNR $>6$ dB and more noise bands or background noise; “Ugly” means SNR $<6$ dB. Energy bars are in jet color scale where redder is more energy and bluer is less energy.

To demonstrate the capabilities of this new *Bayesian VMD Method*, three examples of different quality audio data files were chosen to represent the variety of results. These three examples are described as “good”, “bad”, and “ugly” with regard to their SNR and difficulty to be visually classified in spectrograms. It should be noted that the data segments in Fig. 2 are isolated from the full audio files that contain them so that the length of the very short pulses can be better visualized.

#### The good

In the “good” example, the less-than-a-millisecond broadband buzz at 1.8 s is the only biological signal in the file. The electrical noise bands, like at 10, 12, 14, 20, and 30 kHz are faint, and there is very little background noise from things like ships, singing baleen whales, seals, storms, or ice, so it is a relatively “clean” file. Using visual inspection, like the manual analysis, it is quite clear that there are peaks in the frequencies associated with Lo pulsed signals. One of these characteristics is the strong red bands centered at about 26.6 and 33.7 kHz. The *VMD-gram* merely accentuated these peaks. The *Bayesian VMD Method* classifier agreed with this manual analysis, which means that it should be able to achieve comparable classification results for pulsed signals of similar length and SNR.

#### The bad

Before isolating the biological signal present between 3.7 and 3.9 s, this “bad” example differentiated itself from the “good” example by having more prominent electrical noise bands (the red horizontal lines every 4.17 kHz) present in the spectrogram. In terms of the biological signals themselves, there are strong peaks around 31, 35.5, and 37 kHz as well as faint peaks around 25.7 and 26.8 kHz. While the peak around 31 kHz is associated with Gg pulsed signals, the 35.5 kHz peak is a strong indicator of Lo pulsed signals^15^. The strongest peak around 37 kHz is indicative of both Gg and Lo pulsed calls^15^, so the species classification remains unclear. This signal was difficult to identify visually, but by including species-specific notches in the manual analysis, Lo was chosen as the most likely species for this pulsed signal. The VMD feature extractor disagreed with this, but the weighted values were very similar: 0.608 for Gg versus 0.604 for Lo. The *VMD-gram* showed that peaks around 25 kHz (indicative of Lo) were obscured by electrical noise in the spectrogram. This remains a “bad” example, though, because it is possible the acoustic propagation is different in the Arctic than in the Eastern Tropical Pacific where Soldevilla *et al*.^15^ worked to develop the peak and notch pattern differences. It is also possible that the animals call differently in the two places, meaning the peak and notch patterns could be different in this dataset than in theirs. Regardless of whether the manual classification is correct, the *Bayesian VMD Method* still determined the pulsed call was more likely from one species over another, albeit barely so, using the work from Soldevilla *et al*.^15^ as ground truth.

#### The ugly

The “ugly” example has the worst combination of factors that make manual analysis using a spectrogram difficult: the overall quality of the signal itself is poor and has low SNR, the electrical noise bands are present, and there is some noise in the lower frequencies of the file. Whereas the “good” and “bad” examples still had at least a 6 dB SNR that would have let them pass the typical quality controls most researchers put in place, the seafoam and yellow colors that are the average in Fig. 2c, f makes it almost impossible to see the signal in orange between 0.05 and 0.15 s. In addition to this low SNR, the electrical noise bands, if not removed from the file, could easily become peaks in the *Bayesian VMD Method* that are not related to delphinid pulsed calls at all.

While barely visible over background noise to the eye, there are energy peaks at approximately 38 kHz and in the low 20 kHz. This is not very helpful since both peaks could be evidence for either Gg or Lo. The *Bayesian VMD Method*, however, was able to make more sense of the “ugly” example for the human eye than the spectrogram could. It amplified the peak around 38 kHz and unveiled a peak at about 34 kHz. This latter frequency is associated with Lo pulsed signals^15^, allowing for a classification to be made. This shows that the *VMD-gram* is able to uncover information that would normally be hidden by a substantial amount of noise when using a spectrogram. Most acoustic studies institute quality control rules, and this file would fail to pass them, thus getting removed from any dataset that it would be a part of. For sparse datasets, removing files because of poor quality is an impairment to the study, so having a method that could retain files by increasing their visualization quality would be helpful.

## Discussion

As climate change occurs, animals that usually occupy temperate habitats shift further towards the poles (a.k.a. northward habitat expansion)^30–32^, so tracking Lo, Gg, and other delphinid, pulsed-signal-producing species will help address crucial ecological concerns about the restructuring of the Arctic food web. Therefore, we must be able to differentiate between similarly calling species like Lo and Gg to understand how quickly food webs are shifting. Timely analysis of acoustic data will enable conservation efforts to respond more quickly. An algorithm that can eliminate background noise, emphasize frequency content in short signals, and use limited acoustic features to differentiate between species while being computationally efficient would advance the processing of marine mammal signals needed to track habitat expansion of species as the climate changes. Adding a visualization tool to improve manual analysis of pulsed calls would also expedite bioacoustical data processing.

**Figure 3:**
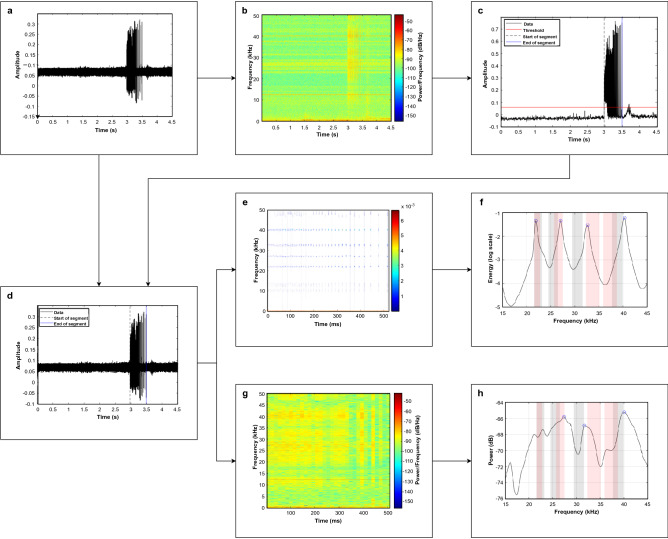
Flowchart demonstrating the usage of the *VMD-gram*. (**a**) The time-domain of the input signal, (**b**) the spectrogram of the audio file, (**c**) application of the threshold and defining time bounds, (**d**) isolating the significant signal component of the input signal using the time bounds from (**c**), (**e**) the *VMD-gram* of the segment, (**f**) the comparison of the segment to Soldevilla et al. 2008, (**g**) the spectrogram of the segment, and (**h**) the comparison of the segment to Soldevilla et al. (2008). Bars in (**f**) and (**h**) indicate the peaks in pulsed signals as documented by Soldevilla et al. (2008) for Gg (gray) and Lo (red).

Our *Bayesian VMD Method* and *VMD-gram* meet these advantageous requirements. They were tested on a difficult dataset—one that was sparse, void of whistles, lacked contextual clues beyond 4.5 s, and had many low SNR files. These difficulties are the reality for much underwater acoustic data, but future work includes testing this new method on a set of more robust (higher SNR) datasets to determine how well our results generalize. Fig. 3 demonstrates the detection of an Lo signal and the usage of the *VMD-gram* in manual analysis. The promise of the *Bayesian VMD Method* is that it achieved recall, precision, and accuracy values similar to previous work in automated detection and classification while differentiating between two species that produce very similar pulsed signals. The EMD detector from previous work^24^ avoided picking most energy peaks generated by noise, but it lacked adaptability and simplicity since multiple tuning parameters had to be manually adjusted for audio files with different levels of background noise. The new detector used in this paper not only automatically estimates the noise floor of each file as an adaptive threshold, but also groups buzzes or clicks based on the inter-click interval of signals of interest (see Fig. 3a–c). Lo and Gg were a prime pair of dolphins to test the *Bayesian VMD Method* on because they produce fewer whistles than other species^13^ and the peak and notch patterns of their pulsed calls are not stereotyped across individuals or geography, yet are two important animals to track in the changing Arctic Ocean.

Successful analysis software today perform detection and classification on sparse data because processing is in real-time (like PAMGuard). For sparse data to give reliable real-time results, a few key features in a single event need to be capitalized on and we can not rely on extracting many features in terabytes of data that have been archived for years. Our application of VMD is essentially a distillation process: a small dataset of the good, the bad, and the ugly SNR quality recordings, using a few key features, can still be analyzed equally well between an algorithm and a human analyst. It is not without its needed improvements, though. The *Bayesian VMD Method* may fail when noise (electrical noise, boat noise, etc.) in a recording is stronger than the desired acoustic signals. To improve it, noise with high power could be removed first to get rid of the bulk of background noises. Additionally, the number of IMFs that the signal is decomposed into could be set adaptively depending on the input acoustic file. One difficulty in making the *Bayesian VMD Method* was establishing the best number of IMFs because 9, what we settled on, was not perfect for every file. Any algorithm or software package that can reliably discern clicks of different species across all the oceans would be a powerful tool in cetacean conservation efforts, and is thus the reason many scientists are focused on advancing such signal processing methods in bioacoustics today. The *Bayesian VMD Method* and *VMD-gram* are two pieces in that pursuit.

## Methods

### Manual analysis

Data files from previous work^2,33,34^ that were manually analyzed (visually validated) and found to contain Lo or Gg pulsed signals were used to test the *Bayesian VMD Method*. No animals were directly involved in this study as all acoustic data were collected passively. Data were collected with passive acoustic listeners (PALs)^35^ at a 100 kHz sampling rate with an adaptive duty cycle between 0.75% and 3.75% depending upon whether the PAL’s software had detected a signal of interest or not^36^. This created up to twenty-one 4.5 s files per day over each yearlong deployment. Visual validation was done using spectrograms generated by Ulysses software (written by A. Thode; optimized by J. Sarkar). The dataset we used in this study only contained clicks or burst pulses except for two files with faint whistles that were not coincident with pulsed signals, so whistles were not a viable signal for contextual use like in the BANTER^13^ study. We used the peak and notch patterns from Soldevilla *et al*. (2008) since they are the best documented pulsed signal characteristics for Gg and Lo and because the geographical range between the ETP groups and the individuals spreading into the Arctic Ocean might overlap.

We used all files that were of good enough quality to manually determine peak and notch patterns to ascribe to a species as well as files from the same day with similarly looking, but poor quality, pulsed signals. The poor quality files as stand-alone signals did not pass quality control guidelines put in place in previous work^2,24,33,34^, but they were recorded close in time to good quality files with more certain IDs. Therefore, they were assigned the same species as a good quality signal.

Because it is known that these two species travel in pods^15^ and mill around while feeding, it was reasonable to assume that pulsed signals close in time to one another were from the same species. These poor-quality files were included in the dataset for VMD accuracy testing to find the point where the *Bayesian VMD Method* failed. If a dataset existed with many good-quality files, then rules set forth by Kowarksi et al.^37^ could have been implemented, but a sparse dataset does not have the advantages of “Big Data”. Poor quality pulsed signals could have been received from the side of the dolphins’ heads or from a distance, so are highly attenuated and variable in both spectral and temporal characteristics^13,38^. It was expected that these poor-quality files would drive down the accuracy of the *Bayesian VMD Method*, but poor quality files are a reality in underwater bioacoustics and manual analysts sift through them regularly, so including them was a better approximation of reality.

For this study, additional manual analysis included visually determining the peak and notch patterns in (1) noisy spectrograms, (2) de-noised spectrograms, and (3) the new *VMD-grams*. If IDs from the three visualizations differed in whether a pulsed signal was “Gg”, “Lo”, or “too difficult to determine”, the ID that was the same for two of the three methods was used as the final ID for the file. These final manual analysis IDs were used as the ground truth set to compare the *Bayesian VMD Method* results against for accuracy and precision/recall calculations. We decided not to use the anecdotally easier *VMD-grams* for the ground truth IDs because using spectrograms for manual analysis has been the historical way to do manual detection and classification work. Note from the results, though, that using the *VMD-gram* IDs as the ground truth improved accuracy. This is because the *VMD-gram* displays the peak and notch patterns more cleanly, thus removing much of the guessing that the human analyst has to do about where the exact peak frequencies appear to be in spectrograms. Finally, the VMD classifier was chosen because previous work^24^ using EMD to distinguish the acoustic signals of Gg from Lo were unsuccessful. Both species’ pulsed signals resulted in the same “EMD identity” labels, meaning they were assigned to the same class. VMD, however, captured the peak and notch patterns of the two species and made it possible to distinguish between them.

**Table 2:** Mean, standard deviation (*SD*), and percent of occurrence (*O*) for the peak and notch patterns established by Soldevilla et al.^15^. Adapted from *Test data* part of their Table IV.

Class	Parameters	Peaks #				Notches #		
		1	2	3	4	1	2	3
Gg	*Mean* (*SD*)	22.4 (0.8)	25.5 (1.0)	30.5 (1.1)	38.8 (1.1)	19.6 (1.3)	27.7 (1.1)	35.9 (1.2)
	*O* %	72	45	82	48	46	64	54
Lo	*Mean* (*SD*)	22.2 (0.6)	26.6 (0.9)	33.7 (1.4)	37.3 (1.4)	19.0 (1.1)	24.5 (0.9)	29.7 (1.4)
	*O* %	89	76	45	62	51	75	66

## Detection and classification system

The *Bayesian VMD Method* we developed can classify pulsed signals with similar frequency content in poor SNR files from underwater acoustic recordings. The Method consists of two parts. The first part scans the incoming audio data as segments that potentially contain signals of interest by detecting energy peaks. It then uses the start and end of the energy peaks to isolate those areas of interest from non-signal areas of the audio file. The second part classifies the detected signals into separate categories based on their frequency content. The algorithms of our Detector and Classifier steps are self-developed, but some key components in them were inspired by previous work^39–41^.

### Detector

The proposed detector uses full audio files that are 4.5 s long at a sampling rate of 100 kHz. It then finds audio file segments where potential signals of interest exist.

For a given audio file, denoted by ${\hat{x}}(n)$, where $n=1, \dots , N$, and N is the total number of samples, the Laplacian Differential Operator (LDO) is applied to ${\hat{x}}(n)$ resulting in an enhanced version of the audio file denoted by *y*(*n*), as follows:

1 $$\begin{aligned} y(n) = \frac{1}{4}\frac{\partial ^2 {\hat{x}}}{\partial n^2} \end{aligned}$$

The LDO enhances the transient signals (edge detection) and filters out the low frequencies ($< 10$ kHz) which are not needed for Gg and Lo pulsed signal classification. The *y*(*n*) is then transformed into a time-frequency representation using Short-time Fourier transform (STFT). The STFT was implemented on 1024 samples with 90% overlap and a 1024-point Hanning window. The magnitude of the STFT matrix *s*(*n*, *f*) is given as ${\hat{S}}(n,f)$.

2 $$\begin{aligned} {\hat{S}}(n,f) = \begin{bmatrix} |s_{11}| &{} \dots &{} |s_{1N}|\\ \vdots &{} \ddots \\ |s_{M1}| &{} &{} |s_{MN}| \end{bmatrix} \end{aligned}$$

Where $N$ is the length of the input segment and $M$ is the number of frequency bins. The dimensionality of matrix ${\hat{S}}(n,f)$ is reduced from 2-D to 1-D as follows:

3 $$\begin{aligned} S_{d}(n) = \sum _{f=1}^{M} {\hat{S}}(n,f) \end{aligned}$$

The resulting temporal sequence is an accumulated sum of all frequency bins from $$\begin{aligned} {\hat{S}}(n,f) \end{aligned}$$, so scaling is applied, as follows:

4 $$\begin{aligned} S_{d}(n) = \frac{S_{d}(n)}{max\{S_{d}(n)\}} \end{aligned}$$

After finding $S_{d}(n)$ from Eq. (4), the mean of $S_{d}(n)$ is subtracted. Then, to determine the boundaries of the acoustic signal, an adaptive threshold is applied. The first step in developing the threshold is to vectorize the matrix ${\hat{S}}(n,f)$ in column order into a vector called $S_{r}(n)$:

5 $$\begin{aligned} S_{r}(n) = \overrightarrow{{\hat{S}}(n,f)} \end{aligned}$$

Then, $S_r (n)$ is scaled similar to $S_{d}(n)$ and is sorted into ascending order, denoted by ${\hat{S}}_r(n)$. The changing point where the root-mean-square level of the sorted curve ${\hat{S}}_r(n)$ changes the most is obtained by minimizing Eq. (6)^39,40,42^

6 $$\begin{aligned} J(k) = \sum _{i=1}^{k-1} \Delta ({\hat{S}}_{r,i}; \chi ([{\hat{S}}_{r,1} \dots {\hat{S}}_{r,k-1}])) + \sum _{i=k}^{N} \Delta ({\hat{S}}_{r,i}; \chi ([{\hat{S}}_{r,k} \dots {\hat{S}}_{r,N}])) \end{aligned}$$

where $k$ and *N* are the index of the changing point and the length of the sorted curve ${\hat{S}}_r (n)$, respectively, and

7 $$\begin{aligned} \sum _{i=u}^{v} \Delta ({\hat{S}}_{r,i}; \chi ([{\hat{S}}_{r,u} \dots {\hat{S}}_{r,v}])) = (u-v+1)\log \left( \frac{1}{u-v+1}\sum _{n=u}^{v}{\hat{S}}_{r,n}\,^{2}\right) \end{aligned}$$

The threshold, $\lambda$, is the value of ${\hat{S}}_r (k)$ which equals the noise floor estimation, and can be represented as follows:

8 $$\begin{aligned} \begin{aligned} {\mathcal {H}}_{0}: S_d(n) < \lambda \\ {\mathcal {H}}_{1}: S_d(n) \ge \lambda \end{aligned} \end{aligned}$$

where ${\mathcal {H}}_0$ and ${\mathcal {H}}_1$ are the hypothesis that the activity was below or above the threshold, respectively. The calculated threshold can vary for each file, thus making it adaptable if ambient noise conditions change between files. The threshold $\lambda$ is then projected onto the temporal sequence $S_{d}(n)$ to extract the boundaries of the regions of the acoustic signal that comprised the detected energy peak. The start and end points of each acoustic signal are determined as the first and last points that are greater than $\lambda$ in amplitude.

**Figure 4:**
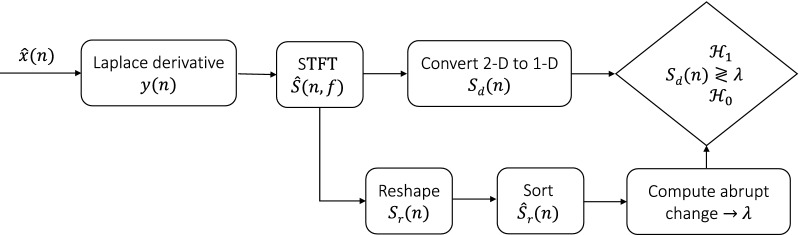
Block diagram of the proposed detector.

The boundaries of the detected segments are scaled by the sampling rate to obtain start and end times which will be used to extract the audio file segments from the original data file in the classification step. Figure 4 illustrates the layout of the the proposed detector.

### Classifier

Once segments with energy peaks were identified, they were scanned by the team’s bioacoustics expert, and any segments confirmed to contain only Gg or Lo signals were sifted out for use in testing the accuracy of the *Bayesian VMD Method* classifier.

In this paper, the metric *weight* was defined for classification purposes. The *weight* for a parameter $\varvec{\theta _i}$ given its measurement $\varvec{y_i}$ is defined as

9 $$\begin{aligned} w(\varvec{\theta _i} \mid \varvec{y_i}) = P_{\varvec{\Theta \mid Y}}(\varvec{\theta _i} \mid \varvec{y_i}) * \varvec{p_i} \end{aligned}$$

where $\varvec{\theta _i}$ is the probability density function (*PDF*) of $\varvec{y_i}$, $\varvec{y_i}$ is one measurement in the measurement vector $\varvec{y}$, $P_{\varvec{\Theta \mid Y}}(\varvec{\theta _i} \mid \varvec{y_i})$ is the posterior probability of the parameter $\varvec{\theta _i}$ given the measurement $\varvec{y_i}$, and $\varvec{p_i}$ is the scaled prominence value of $\varvec{y_i}$.

When a detected audio file segment is fed into the *Bayesian VMD* classifier, the classification process starts with a feature extraction step. During this step, peak and notch frequencies and their prominence values were obtained from the VMD-Hilbert spectrum of the segment. The prominence measures how much a peak stands out due to its intrinsic height or how much a notch stands out due to its depth and its location relative to surrounding peaks or notches. In general, peaks that are taller and more isolated have a higher “prominence” (p) than peaks that are shorter or surrounded by other peaks.

In the feature extraction step, VMD decomposed the input audio segment into a set of IMFs. The HHT was then applied to all IMFs to create a Hilbert spectrum with a frequency resolution of 50 Hz. The Hilbert spectrum is a matrix, $H(n,f)$ that contains the instantaneous energies, $h(n,f)$.

10 $$\begin{aligned} H(n,f) = \begin{bmatrix} h_{11} &{}\dots &{} h_{1R} \\ \vdots &{} \ddots \\ h_{Q1} &{} &{} h_{QR} \end{bmatrix} \end{aligned}$$

where r is the length of the input segment and q is the number of frequency bins in $H$.

The matrix $H (n,f)$ is then converted from a 2-D array to a 1-D spectral representation by summing all instantaneous energy values in each frequency bin, as follows:

11 $$\begin{aligned} H(f) = \sum _{n=1}^{R} H(n,f) \end{aligned}$$

**Figure 5:**
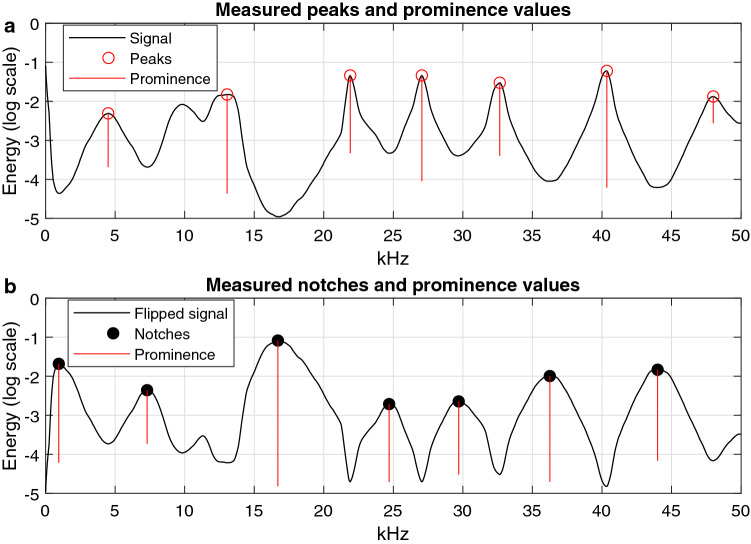
Example of locating peak and notch frequencies and how prominent they are compared to other peaks and notches. The wave form in (**a**) is the smoothed energy summation sequence from the Hilbert spectrum of the Lo signal in Fig. 1. Subplot (**b**) is a flipped version of the energy summation sequence for the convenience of extracting notch frequencies and their prominence values. The length of the red line represents the prominence value of a peak or notch.

The energy summation sequence was converted to a base-10 logarithmic scale and then smoothed by passing through a 17-point median filter and an 11-point moving average filter for the purpose of easily extracting features. All peaks and notches in the sequence whose prominence values exceeded the threshold of 0.5 were located, and their frequency values and prominence values were then stored as extracted features from the input signal (see Fig. 5).

For testing the effectiveness of the VMD feature extractor, a second set of features were extracted from the FFT-based power spectrum using the same input signals with the Welch’s algorithm. The FFT-based spectrum was calculated on 2048 samples with 50% overlap and a 2048-point Hanning window with 48.82 Hz frequency resolution. The power spectral density sequence was then converted to dB and went through a 21-point median filter and a 15-point moving average filter. Feature extraction followed the same strategies as in VMD feature extractor except using a prominence threshold of 2 dB.

Next, the measured features, frequencies (Hz) of the peaks and notches (henceforth referred to as “measured peaks and notches”), were matched with the probability distribution functions (PDFs) of peaks and notches (henceforth referred to as “parameter peaks and notches”) from Soldevilla *et al*. (2008). The matching between measured and parameter peaks and notches was done in preparation of weight calculations, and it was implemented for both Gg and Lo. There are four Gaussian PDFs for parameter peaks and three for parameter notches for each species in Soldevilla *et al*. (2008) (Table 2). A 95% confidence interval of a Gaussian PDF was used here as a frequency range defined as 1.96 standard deviations to the left and right of its mean value. When measured peaks and notches were matched to parameter peaks and notches, only the peak or notch that fell within a 95% confidence interval were kept. Any peaks or notches outside the 95% confidence intervals were discarded.

Because there are overlaps between the 95% confidence intervals of 22.4 kHz and 25.5 kHz parameter peaks of Gg and between 33.7 kHz and 37.3 kHz parameter peaks of Lo (see Table 2), it is likely that some measured peaks will fall in the overlapping areas. In this paper, the maximum a posterior (MAP) estimation^41^ was used to determine which PDF results in the measured peak in an overlapping area. For a measured peak that falls into an overlapping area, two parameter peaks’ PDFs are plugged in the MAP estimation equation sequentially, and then the measured peak will be matched with the PDF that maximizes the posterior probability of it given the measured peak.

After the preliminary match, if more than one measured peak or notch remains in any one PDF confidence interval, the measured peak and notch with the highest prominence value is selected as the real measured peak or notch of this PDF, and the redundant ones are discarded. Finally, all remaining peak prominence values and notch prominence values were scaled to be between 0 and 1, respectively.

Once peak and notch matching and selection was finished, Bayesian weights were calculated to select the most likely species. From Bayes’s rule, the posterior probability of a parameter given its measurement is proportional to the product of the likelihood function of the measurement given the parameter and the prior probability of the parameter^41^, as shown in Eq. (12).

12 $$\begin{aligned} P_{\varvec{\Theta \mid Y}}(\varvec{\theta _i} \mid \varvec{y_i}) \propto f_{\varvec{Y \mid \Theta }}(\varvec{y_i} \mid \varvec{\theta _i}) P_{\varvec{\Theta }}(\varvec{\theta _i}) \end{aligned}$$

therefore, substitution of the posterior probability in Eq. (9) yields

13 $$\begin{aligned} w(\varvec{\theta _i} \mid \varvec{y_i}) = f_{\varvec{Y \mid \Theta }}(\varvec{y_i} \mid \varvec{\theta _i}) *P_{\varvec{\Theta }}(\varvec{\theta _i}) * \varvec{p_i} \end{aligned}$$

**Figure 6:**
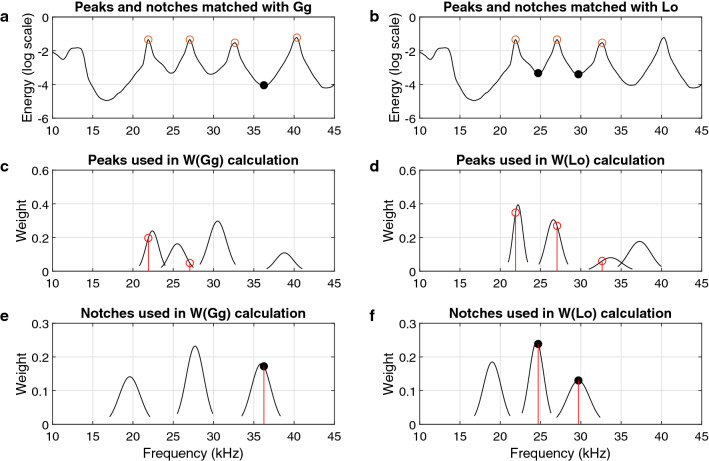
Example of feature matching. The top plots show a set of measured peaks and notches matched with both Gg’s PDFs (**a**) and Lo’s PDFs (**b**) parameter peaks and notches like in Fig. 5 during the feature matching and selection step. Middle plots show how closely to the parameter PDFs that the measured peaks match either Gg (**c**) or Lo (**d**) and their weight calculations. The width of each PDF represents its 95% confidence interval, and the ordinate represents the weight value. Subplots (**e**) and (**f**) show the same weight calculations for notches. The final weight value is the summation of all weight values of peaks and notches matched with Gg or Lo.

With all PDFs and *a priori* probabilities from Soldevilla *et al*. (2008), the weight value in terms of Gg and Lo given a set of measurements, $\varvec{y}$, was obtained by Eqs. (13) and (14)

14 $$\begin{aligned} w(Gg \mid \varvec{y}) = \sum _{\forall i} w(\varvec{\theta _i} \mid \varvec{y_i}) \qquad w(Lo \mid \varvec{y}) = \sum _{\forall j} w(\varvec{\theta _j} \mid \varvec{y_j}) \end{aligned}$$

where $\varvec{y_i}$ and $\varvec{y_j}$ are the remaining measured peaks and notches that were matched with Gg’s PDFs and Lo’s PDFs after the matching and matching step. The feature matching and selection results and the weight calculation process are shown in Fig. 6.

**Figure 7:**
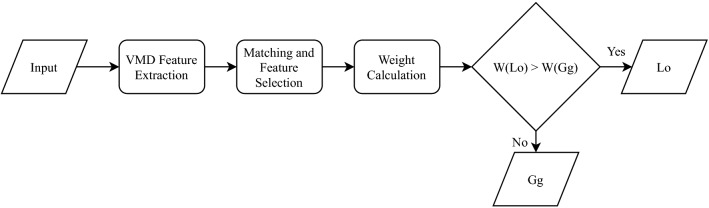
Block diagram of the *Bayesian VMD Method* classifier.

The last step was a comparison between weight values in terms of Gg and Lo. If $w(Lo \mid \varvec{y}) > w(Gg \mid \varvec{y})$, the signal was labeled an Lo signal; otherwise, it was labeled a Gg signal. The classifier is illustrated in Fig. 7. The weight values are significant to three digits because weights are normally smaller than 1.000 and three significant digits was sufficient for comparing all calculated weight values for these audio files. In the case that the weight comparison is equal to three significant digits (even though this never happened in these 174 signals), the Bayesian VMD algorithm will automatically classify the input as a Gg signal given that the highest precision (85.91%) by the *Bayesian VMD Method* was achieved on Gg.
